# Short‐term arm immobilization modulates excitability of inhibitory circuits within, and between, primary motor cortices

**DOI:** 10.14814/phy2.15359

**Published:** 2022-06-27

**Authors:** Erin M. King, Lauren L. Edwards, Michael R. Borich

**Affiliations:** ^1^ Neuroscience Graduate Program Graduate Division of Biological and Biomedical Sciences Emory University Atlanta Georgia USA; ^2^ Department of Rehabilitation Medicine Emory University Atlanta Georgia USA; ^3^ Department of Biomedical Engineering Georgia Institute of Technology Atlanta Georgia USA

## Abstract

Previous research has suggested that short‐term immobilization of the arm may be a low‐cost, non‐invasive strategy to enhance the capacity for long‐term potentiation (LTP)‐like plasticity in primary motor cortex (M1). Short‐term immobilization reduces corticospinal excitability (CSE) in the contralateral M1, and interhemispheric inhibition (IHI) from ipsi‐ onto contralateral M1 is increased. However, it is unclear whether reduced CSE and increased IHI are associated with changes in intracortical inhibition, which has been shown to be important for regulating neuroplasticity in M1. The current study used transcranial magnetic stimulation to evaluate the effects of short‐term (6 h) arm immobilization on CSE, IHI, and intracortical inhibition measured bilaterally in 43 neurotypical young adults (23 immobilized). We replicated previous findings demonstrating that immobilization decreased CSE in, and increased IHI onto, the immobilized hemisphere, but a significant change in intracortical inhibition was not observed at the group level. Across individuals, decreased CSE was associated with a decreased short‐interval intracortical inhibition, an index of GABA_A_‐ergic inhibition, within the immobilized hemisphere only in the immobilization group. Previous research has demonstrated that decreases in GABA_A_‐ergic inhibition are necessary for the induction of LTP‐like plasticity in M1; therefore, decreased intracortical inhibition after short‐term arm immobilization may provide a novel mechanism to enhance the capacity for LTP‐like plasticity within M1 and may be a potential target for strategies to augment plasticity capacity to enhance motor learning in health and disease.

## Graphical abstract

Previous research has suggested that short‐term immobilization of the arm may be a low‐cost, non‐invasive strategy to enhance the capacity for long‐term potentiation (LTP)‐like plasticity in primary motor cortex (M1). The current study used transcranial magnetic stimulation (TMS) to evaluate the effects of short‐term (6 h) arm immobilization on M1 excitability and cortical inhibition, in 43 neurotypical young adults. We replicated previous findings demonstrating that immobilization decreased M1 excitability and increased IHI onto, the immobilized hemisphere suggesting that decreased intracortical inhibition after short‐term arm immobilization may provide a novel mechanism to enhance the capacity for LTP‐like plasticity within M1.

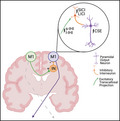

## 1 INTRODUCTION

Experience‐dependent plasticity is a fundamental property of the brain and refers to the ability to undergo structural and functional change in response to experiences (Fu & Zuo, 2011). In sensorimotor networks, experience‐dependent plasticity allows organisms to adjust their behavior to successfully interact with their environment over time. Mechanisms of neuroplasticity have been shown to underpin experience‐dependent learning of new motor skills in rodents (Rioult‐Pedotti et al., 2000) and humans (Butefisch et al., 2000). Additionally, recovery of motor function after neural insult, such as stroke, is influenced by experience‐dependent plasticity (Allred et al., 2014). Given the fundamental importance of experience‐dependent plasticity for brain function and learning, understanding the mechanisms of plasticity, and developing strategies to enhance these mechanisms is important. Synaptic strengthening through long‐term potentiation (LTP) is a main contributor to experience‐dependent plasticity in human motor cortex (Butefisch et al., 2000). However, synaptic strength must be maintained within a target range to prevent over‐ or under‐excitation of the circuit (Abbott & Nelson, 2000). This model of homeostatic metaplasticity suggests that the recent history of synaptic strength influences the degree to which further synaptic strengthening can occur (Bienenstock et al., 1982; Park et al., 2014; Whitt et al., 2014). This concept has been demonstrated in rodent primary motor cortex (M1), where in order to maintain neural activity within a physiologically stable dynamic range, the ability to induce LTP or long‐term depression (LTD) was adjusted by the prior history of synaptic activity associated with motor skill training (Rioult‐Pedotti et al., 2000). Additionally, similar homeostatic mechanisms have been demonstrated indirectly in human M1 using noninvasive brain stimulation (Karabanov et al., 2015; Muller et al., 2007; Ziemann et al., 2004).

A noninvasive approach to experimentally induce experience‐dependent plasticity in M1 is short‐term limb immobilization. Short bouts (<12 h) of arm immobilization reduce input to, and output from, the contralateral sensorimotor cortex, resulting in a transient decrease in corticospinal excitability (CSE) in M1 as measured with transcranial magnetic stimulation (TMS) (Clark et al., 2010; Crews & Kamen, 2006; Facchini et al., 2002; Huber et al., 2006; Karita et al., 2017; Opie et al., 2016). Reduced M1 CSE is thought to reflect a decrease in synaptic strength through LTD‐like mechanisms (Huber et al., 2006). Subsequently, the capacity for synaptic strengthening in M1 is enhanced immediately after immobilization in healthy humans, and the degree of enhanced capacity for plasticity is correlated with the degree of decrease in CSE (Rosenkranz et al., 2014). CSE is influenced by multiple inhibitory and excitatory circuits, made up of several intracortical and interhemispheric inputs onto pyramidal output neurons. Currently, circuit‐specific changes in neurophysiology underlying the increase in markers of systems‐level plasticity after immobilization in humans have not been fully characterized. Previous research has demonstrated that excitability in both excitatory and inhibitory circuits in M1 can be modulated in a homeostatic manner (Murakami et al., 2012); however, it is unclear whether intracortical inhibition contributes to the possible metaplastic effects of short‐term immobilization and whether homeostatic changes in excitatory and inhibitory circuits influence one another.

**FIGURE 1:**
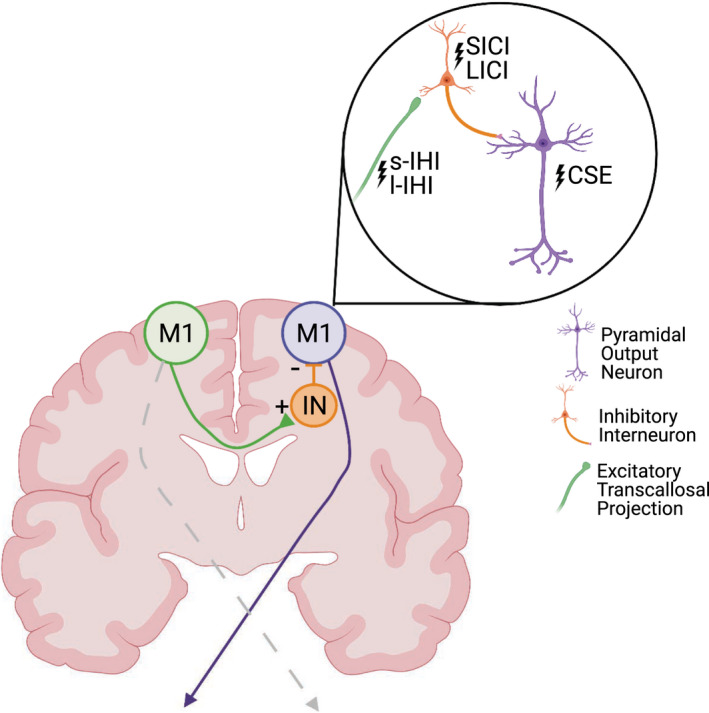
Cortical circuits of interest to evaluate the neuromodulatory effects of short‐term arm immobilization. Activation of neurons in M1 (green) leads to inhibition of contralateral M1 (purple) through excitatory transcallosal projections synapsing onto inhibitory interneurons (orange). Transcranial magnetic stimulation (TMS) can be used to probe different components of this circuit.

When a limb is immobilized, cortical excitability can be modulated bilaterally and is likely mediated to an extent by direct transcallosal projections between homologous cortical regions. Transcallosally‐mediated interhemispheric inhibition (IHI) is generated by excitatory transcallosal projections that synapse onto inhibitory interneurons in the opposite hemisphere (Chen, 2004; Ferbert et al., 1992; Figure 1). Avanzino et al. (2011) demonstrated that IHI from the non‐immobilized M1 onto the immobilized M1 significantly increased after short‐term arm immobilization and IHI from the immobilized M1 onto the non‐immobilized M1 was reduced (Avanzino et al., 2011). Since the IHI circuit is, at least, di‐synaptic, consisting of both excitatory and inhibitory components, it remains unclear whether immobilization modulates activity of the excitatory transcallosal projections or the inhibitory interneurons receiving transcallosal input.

Several studies have measured intracortical inhibition after a period of immobilization, but results have been inconsistent (Clark et al., 2010; Opie et al., 2016; Rosenkranz et al., 2014). Short‐interval intracortical inhibition (SICI), an index of GABA_A_‐ergic processes and assessed with paired pulse TMS (inter‐pulse interval range: 1–5 ms), has been found to be decreased (Rosenkranz et al., 2014) or unchanged (Clark et al., 2010; Opie et al., 2016) after immobilization. Long‐interval intracortical inhibition (LICI, inter‐pulse interval range: 50–200 ms), an index of GABA_B_‐ergic processes, has been shown to be increased during muscle contraction following of 3 weeks of immobilization (Clark et al., 2010) but unchanged when measured at rest (Clark et al., 2010; Opie et al., 2016).

Understanding the effect of experimental manipulations of neural activity in humans, such as immobilization, on inhibitory circuit activity is important for identifying mechanisms of enhanced plasticity. In particular, GABA‐mediated neuroplasticity is a fundamental contributor to motor skill learning and can alter the functioning of entire neural circuits (Butefisch et al., 2000; King et al., 2020; Kolasinski et al., 2019). Modulation of GABAergic inhibition is essential for synaptic plasticity in primary sensorimotor cortex in humans (Floyer‐Lea et al., 2006; Kim et al., 2014) and rodents (Kida et al., 2016). However, it remains unclear how inter‐ and intra‐cortical inhibitory circuit activity are independently and/or co‐modulated by experience‐dependent plasticity induction.

The purpose of the current study was to characterize the neuromodulatory effects of short‐term (6 h) arm immobilization on intra‐ and inter‐cortical circuit excitability compared to an equivalent period without immobilization. We used a battery of TMS‐based assessments to evaluate the neurophysiologic effects of immobilization on cortical excitability within and between M1s in neurotypical healthy adults randomly assigned to undergo immobilization or no immobilization. Due to the inherent inter‐individual variability in human neuromodulation studies (Muller‐Dahlhaus et al., 2008; Schilberg et al., 2017), we also examined associations between neurophysiologic outcomes across individuals.

## 2 MATERIALS AND METHODS

### 2.1 Participants

Forty‐four healthy participants (13 male) aged 18–35 years old (mean = 24.5 ± 4.7 years) participated in this study. Inclusion criteria included (1) no history of movement impairment or neurodegenerative disease, (2) right‐handedness according to the Edinburgh Handedness Scale (Oldfield, 1971), and (3) no contraindication to TMS (Rossi et al., 2009). All study procedures were approved by the Emory University Institutional Review Board in accordance with the Declaration of Helsinki. Informed written consent was obtained for each participant. One participant reported taking a nap in between TMS sessions. Due to the potential confounding effect of sleep on neural plasticity (Tononi & Cirelli, 2014), data from this participant were excluded from the analysis.

### 2.2 TMS assessments of intra‐ and inter‐cortical excitability

During testing, participants were seated with arms in a resting position and neutral joint angles. Surface electromyography (EMG) electrodes were placed in a belly‐tendon montage to measure TMS‐motor evoked potentials (MEPs) of the FDI muscles during all TMS assessments. Additionally, a 30 mm plate ground electrode was affixed over the dorsum of the hand. The skin under each electrode was prepared using sandpaper and alcohol wipes to ensure optimal contact between the skin and the electrodes. EMG data were sampled using a 16‐channel bipolar system (BrainAmp ExG amplifier; Brain Products GmbH) at a rate of 5000 Hz, across a range of ±16.384 mV, and band‐pass filtered at 10–1000 Hz.

To identify the cortical motor representation of the FDI, stereotactic neuronavigation (Brainsight®; Rogue Research) was used to register surface head landmarks to an MNI template image. The hand representation in the posterior bank of the precentral gyrus (M1) was identified manually and used as the starting point for motor hotspot identification for the target FDI muscle. Monophasic TMS pulses (Magstim 200^2^; MagStim) were delivered in the posterior–anterior direction using a 70 mm figure‐of‐eight coil. Once an MEP was elicited at a given stimulation intensity, the coil was systematically moved to determine the scalp location resulting in the largest MEP at that intensity. Then, the resting motor threshold (RMT) was determined for the M1 hotspot in each hemisphere using the ML‐PEST method (Awiszus, 2003). Guided real‐time stereotactic navigation was used throughout the sessions to ensure consistent TMS targeting of the FDI representation in M1. TMS was used to evaluate CSE, IHI, and intracortical inhibition in M1 bilaterally both before and after 6 h of immobilization (Figure 1). The *target* hemisphere for all participants was the right M1 corresponding to the left, non‐dominant arm that was immobilized in the Immobilization group. The *non‐target* hemisphere was left M1; corresponding to the right, dominant arm that was not immobilized in either group.

CSE was measured by the average peak‐to‐peak amplitude of the MEP in response to 20 unpaired TMS pulses at 120% of RMT (SP120). Paired pulse (PP) paradigms involved a conditioning stimulus followed by a test stimulus at a given interstimulus interval (see Table 1 for stimulation parameters for each assessment). Conditioning and test pulses were either applied to the FDI representation of the same hemisphere (intrahemispheric) or FDI representations in opposite hemispheres (interhemispheric). The average peak‐to‐peak amplitude of the conditioned MEP response across 20 trials was divided by the average MEP response during the SP120 condition (PP/SP120) to measure the degree of inhibition caused by the conditioning pulse. RMT was reevaluated after the immobilization period, and stimulation intensities for all measures were adjusted accordingly. TMS assessments, summarized in Table 1, were performed in both the target and non‐target hemispheres in a randomized order (Figure 1). Twenty TMS pulses were delivered for each assessment to demonstrate within‐ and between‐session reliability of each measure (Goldsworthy et al., 2016).

**TABLE 1:** Summary of TMS‐based outcome measures to evaluate immobilization effects on cortical excitability

TMS condition	Neural process(es) measured	Paired/unpaired	Conditioning pulse intensity	Test pulse intensity	Interstimulus interval (ISI)
Resting motor threshold (RMT)	Neuronal membrane excitability (34)	N/A	N/A	N/A	N/A
Corticospinal excitability (CSE)	Corticospinal excitability (35)	Unpaired	N/A	120% RMT	N/A
Short‐interval intracortical inhibition (SICI)	Synaptic GABA_A_ (34)	Paired‐intrahemispheric	80% RMT	120% RMT	2 ms
Long‐interval intracortical inhibition (LICI)	Synaptic GABA_B_ (34, 36)	Paired‐intrahemispheric	120% RMT	120% RMT	60 ms
Short interhemispheric inhibition (s‐IHI)	Interhemispheric activation of GABA_A_ (37)	Paired‐interhemispheric	120% RMT	120% RMT	10 ms
Long interhemispheric inhibition (l‐IHI)	Interhemispheric activation of GABA_B_ (38)	Paired‐interhemispheric	120% RMT	120% RMT	50 ms

### 2.3 Arm immobilization

After completion of the morning testing session, 23 (4 male) out of the 43 included participants were randomly assigned to undergo arm immobilization. These individuals were instructed to wear a finger control mitt on the left (non‐dominant) hand that secured positioning of the fingers in a padded mitt to restrict finger movement and sensory input. In addition to the hand mitt, the arm was placed in a sling to reduce movement of the wrist, elbow, and shoulder joints. Participants were instructed to move their left arm as little as possible during the immobilization period but to use the non‐immobilized arm as they would normally. The other 20 (9 male) participants were randomly assigned to be in the No Immobilization control group. These individuals did not wear a mitt or a sling and were told to use their arms normally throughout the day. Activity monitors (wGT3x‐BT; ActiGraph) were worn on both wrists by all participants to measure bilateral arm movements between sessions and determine compliance with the immobilization procedure.

### 2.4 MEP analysis and statistical approach

A custom Matlab (2016a) script was used to automatically identify the minimum and maximum peak amplitudes of the TMS‐evoked response in the FDI to calculate the peak‐to‐peak amplitude for all MEPs. Each MEP was also confirmed visually. Mean MEP amplitude for each TMS assessment was analyzed before and after immobilization, and between‐group analyses were performed to examine changes in excitability between those who were immobilized compared to those who were not immobilized. Mixed‐effects analyses, within‐subject factors of time (AM [pre‐immobilization] or PM [post‐immobilization]) and hemisphere (target or nontarget) were performed for each group (control or immobilization group) to evaluate within‐group effects of immobilization. To assess associations between immobilization effects on different outcome measures within individuals, we performed bivariate correlation analyses with Pearson's correlation coefficients between changes in CSE with changes in interhemispheric and intracortical inhibition, adjusted for multiple comparisons. A mixed‐effects analysis was performed to determine the effects of arm (target or non‐target) and group (control or immobilized) on activity counts and assess compliance with the immobilization protocol. Prism GraphPad 8 statistical packages were used for all statistical analyses. Significance level was set as α ≤ 0.05. Non‐significant *p*‐values between 0.05 and 0.10 were considered non‐significant trends in the data. Data are presented in figures as change for visualization purposes. All data met the assumptions of normality and homogeneity of variance.

## 3 RESULTS

### 3.1 Activity monitoring

**FIGURE 2:**
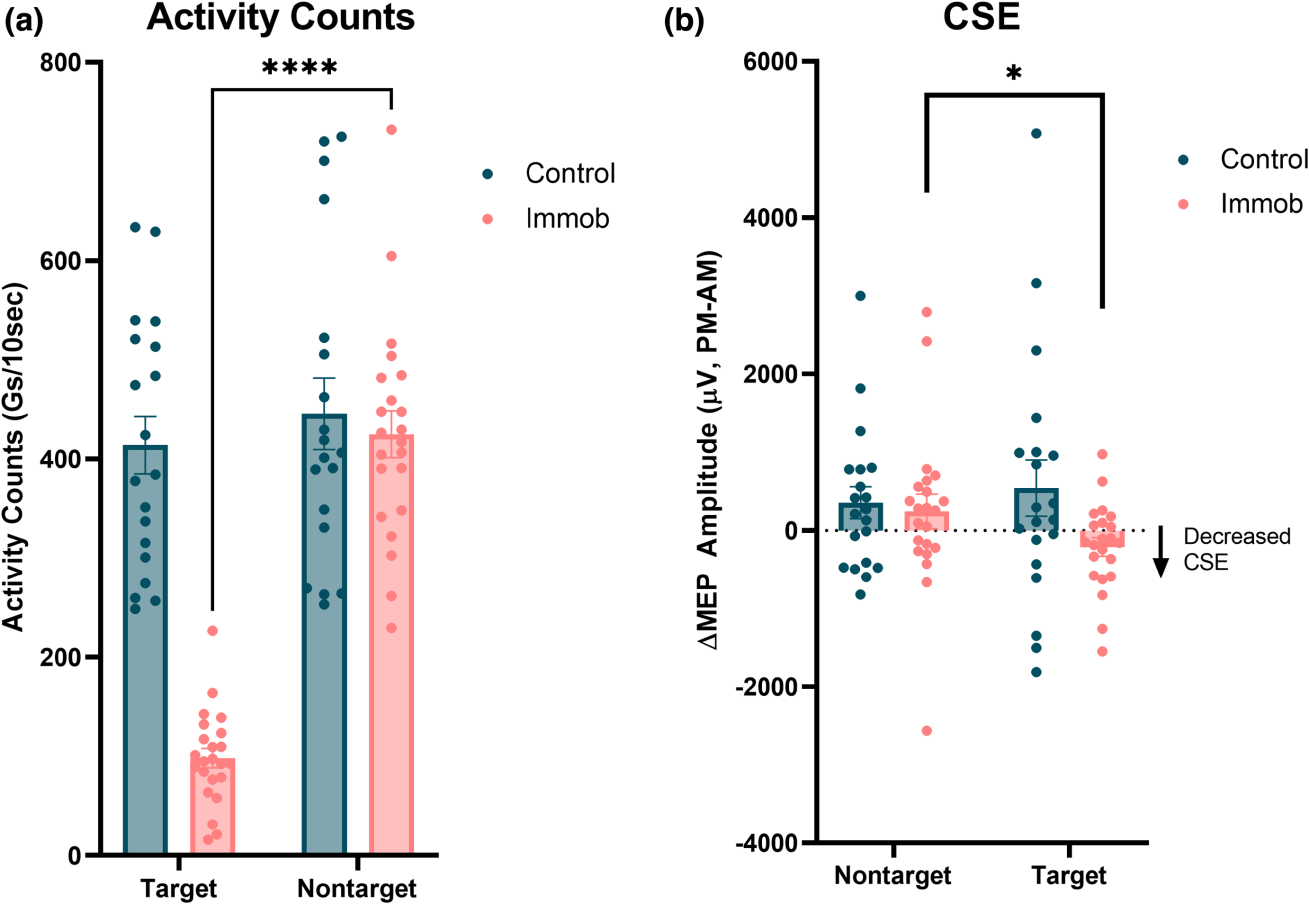
(a) Immobilized participants demonstrated significantly reduced activity in their immobilized (target) arm during the 6‐h immobilization period (****p < 0.0001). (b) Corticospinal excitability (CSE) was significantly reduced following immobilization (*p < 0.05).

A mixed‐effects analysis showed significant effects of arm (*F* = 163.7, *p* < 0.0001) and group (*F* = 26.8, *p* < 0.0001), and there was a significant Group × Arm interaction (*F* = 110.9, *p* < 0.0001) on wrist activity counts (Figure 2a). Sidak's multiple comparisons test demonstrated that the immobilized individuals used their target (immobilized) arm significantly less than their nontarget (non‐immobilized) arm (*t* = 17.2, *p* < 0.0001).

### 3.2 Resting motor threshold (RMT)

A mixed‐effects analysis showed a non‐significant trend for an effect of hemisphere (*F* = 3.9, *p* = 0.054) on RMT, where RMT was higher in the target hemisphere in both groups across testing sessions. Additionally, there was a non‐significant trend for an effect of time (*F* = 3.5, *p* = 0.07), where RMT increased from the AM to the PM session. There was no effect of group (*F* = 1.3, *p* = 0.26) on RMT.

### 3.3 Corticospinal excitability (CSE)

Mixed‐effects analyses demonstrated that CSE significantly increased from AM to PM in the control group (Time: *F* = 4.7, *p* = 0.037), and there was a significant effect of immobilization on the change in CSE in the immobilized group (Group × Time: *F* = 4.4, *p* = 0.04; Figure 2b).

### 3.4 Interhemispheric inhibition (SIHI & LIHI)

**FIGURE 3:**
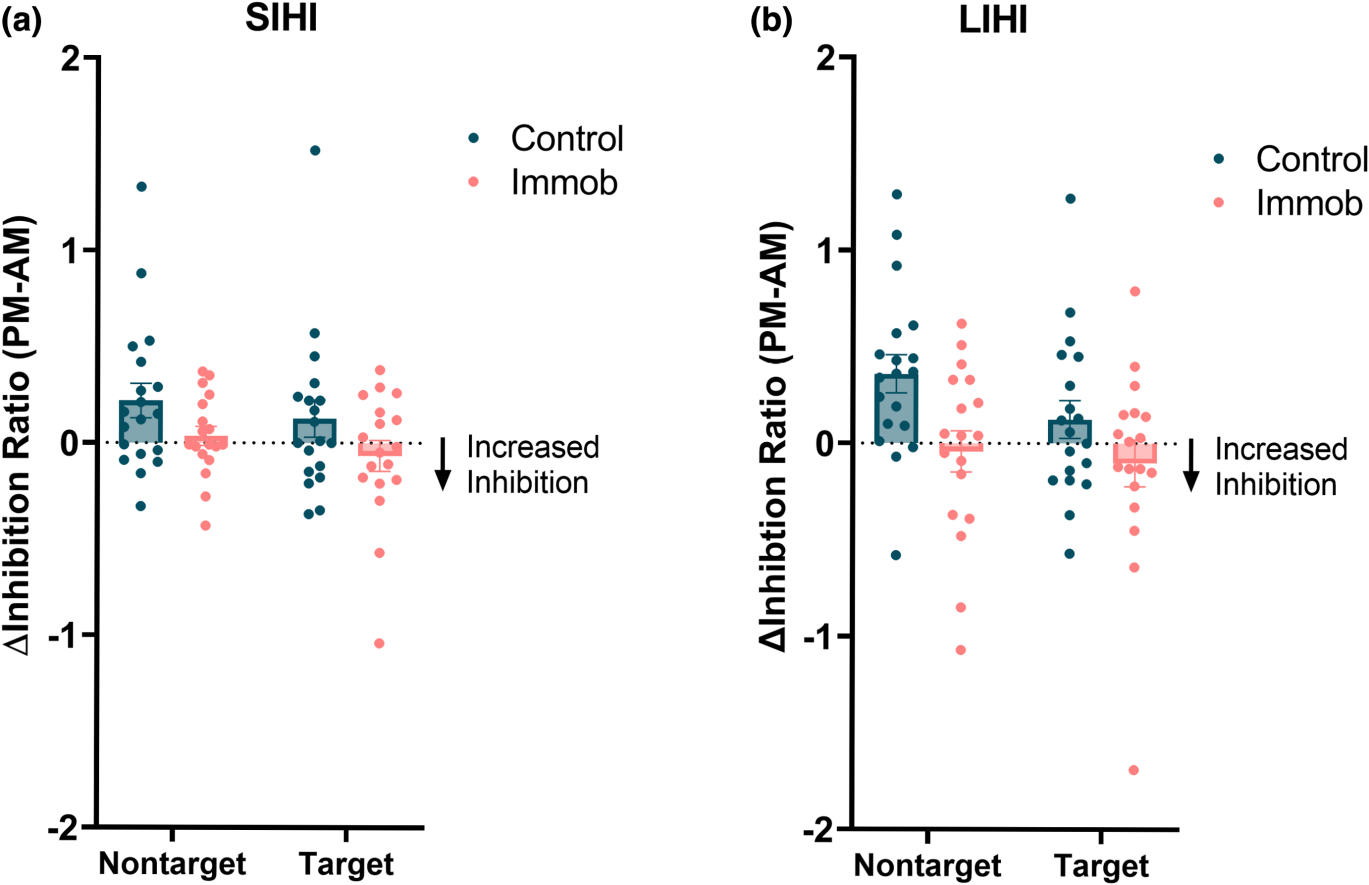
Immobilization led to an increase in (a) short interhemispheric inhibition (SIHI) (*p < 0.05) and (b) long interhemispheric inhibition (LIHI) (**p < 0.01) from the nontarget hemisphere to the target hemisphere.

In both IHI conditions, control participants showed a bilateral release of interhemispheric inhibition over the course of the day, with less IHI observed at the PM session compared to the AM session. However, immobilization led to an increase in inhibition from the nontarget hemisphere onto the target hemisphere. Additionally, the decrease in inhibition from target to nontarget hemisphere observed in controls was blocked for both short interhemispheric inhibition (SIHI) and long interhemispheric inhibition (LIHI) (Figure 3a). Analyses separated by group demonstrated that there was a significant decrease in SIHI in the control group (Time: *F* = 6.8, *p* = 0.01), and SIHI was greater onto the nontarget hemisphere (Hemisphere: *F* = 7.01, *p* = 0.01). In the immobilized group, there was a significant effect of hemisphere with higher IHI onto the non‐target hemisphere across test sessions (Hemisphere: *F* = 19.2, *p* = 0.0001).

For LIHI, a mixed‐effects analysis demonstrated greater IHI onto the nontarget hemisphere (Hemisphere: *F* = 25.2, *p* < 0.0001). Additionally, immobilization significantly modulated the change in LIHI over the course of the day (Group × Time: *F* = 8.8, *p* = 0.006) (Figure 3b). Analyses separated by group indicated that LIHI significantly decreased over the course of the day (Time: *F* = 12.3, *p* = 0.001) and was greater onto the nontarget hemisphere (Hemisphere: *F* = 8.1, *p* = 0.007) in control participants. In the immobilized participants, LIHI was also greater onto the nontarget hemisphere (Hemisphere: *F* = 17.6, *p* = 0.0002).

### 3.5 Intracortical inhibition (SICI & LICI)

**FIGURE 4:**
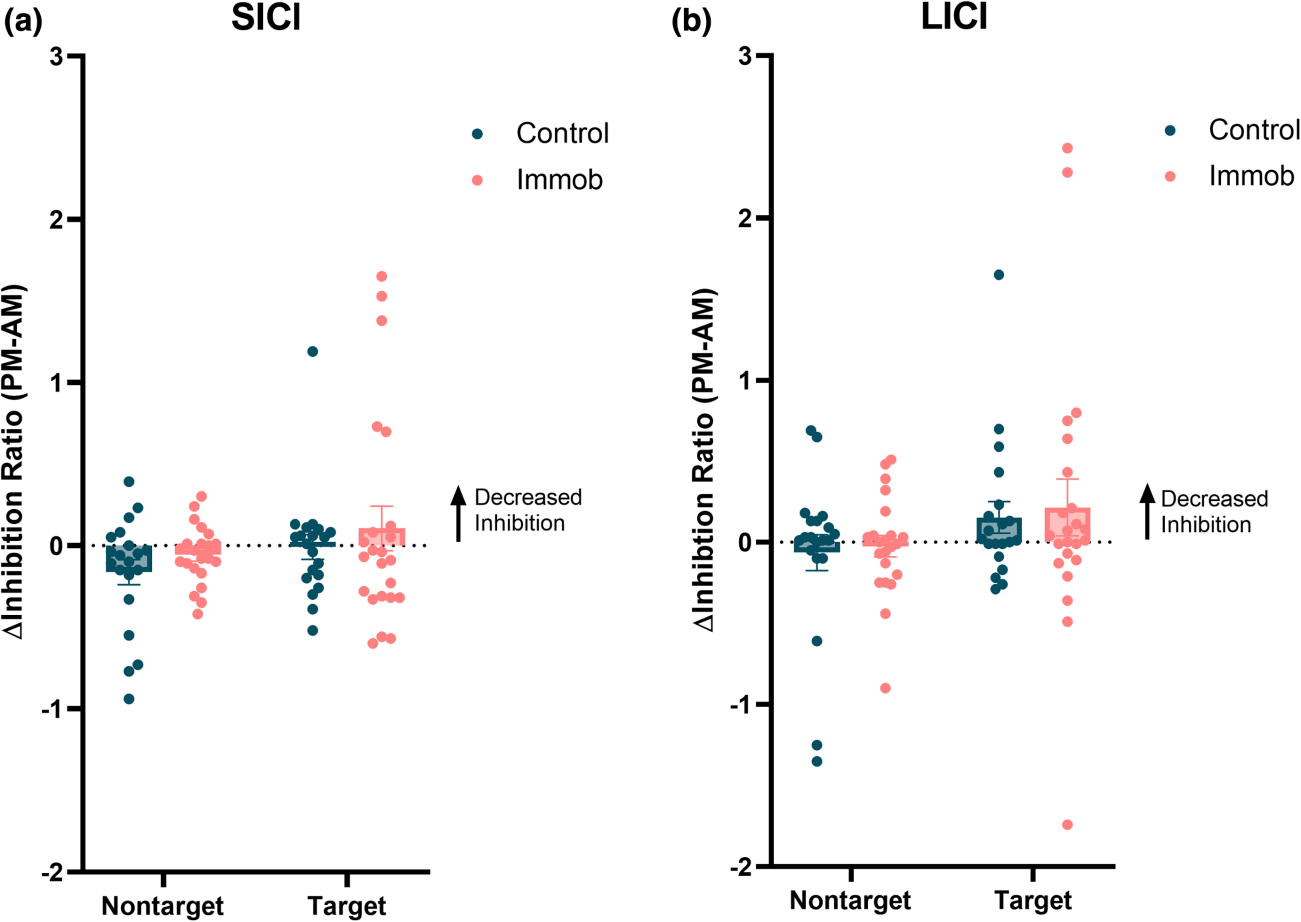
Intracortical inhibitory circuits were not significantly modulated by immobilization. Mixed effects analyses showed no significant effect of immobilization on (a) short‐interval intracortical inhibition (SICI) or (b) long‐interval intracortical inhibition (LICI). Measures of intracortical inhibition showed a non‐significant reduction in inhibition in the target hemisphere following immobilization.

A mixed‐effects analysis showed non‐significant trends for differences in SICI between hemispheres (Hemisphere: *F* = 3.7, *p* = 0.06) and an interaction between time and hemisphere (Hemisphere × Time: *F* = 3.2, *p* = 0.08) on SICI (Figure 4a). Within groups, mixed‐effects analyses demonstrated that SICI was not significantly different between hemispheres (Hemisphere: *F* = 0.22, *p* = 0.64) and did not change throughout the day (Time: *F* = 2.5, *p* = 0.12) in non‐immobilized control participants. In immobilized participants, SICI was not significantly different between hemispheres (Time: *F* = 0.08, *p* = 0.78), but there was a non‐significant trend for lower SICI in the target hemisphere at both time points (Hemisphere: *F* = 3.4, *p* = 0.07).

A mixed‐effects analysis showed that the left, nontarget hemisphere had increased LICI (Hemisphere: *F* = 7.5, *p* = 0.009), and there was a non‐significant trend for an interaction between time and hemisphere (Hemisphere × Time: *F* = 2.9, *p* = 0.09) on LICI (Figure 4b). Mixed‐effects analysis for the control group and the immobilization group separately demonstrated no significant effects of time or hemisphere, as well as no interaction effect, in either group.

### 3.6 Bivariate correlations

**FIGURE 5:**
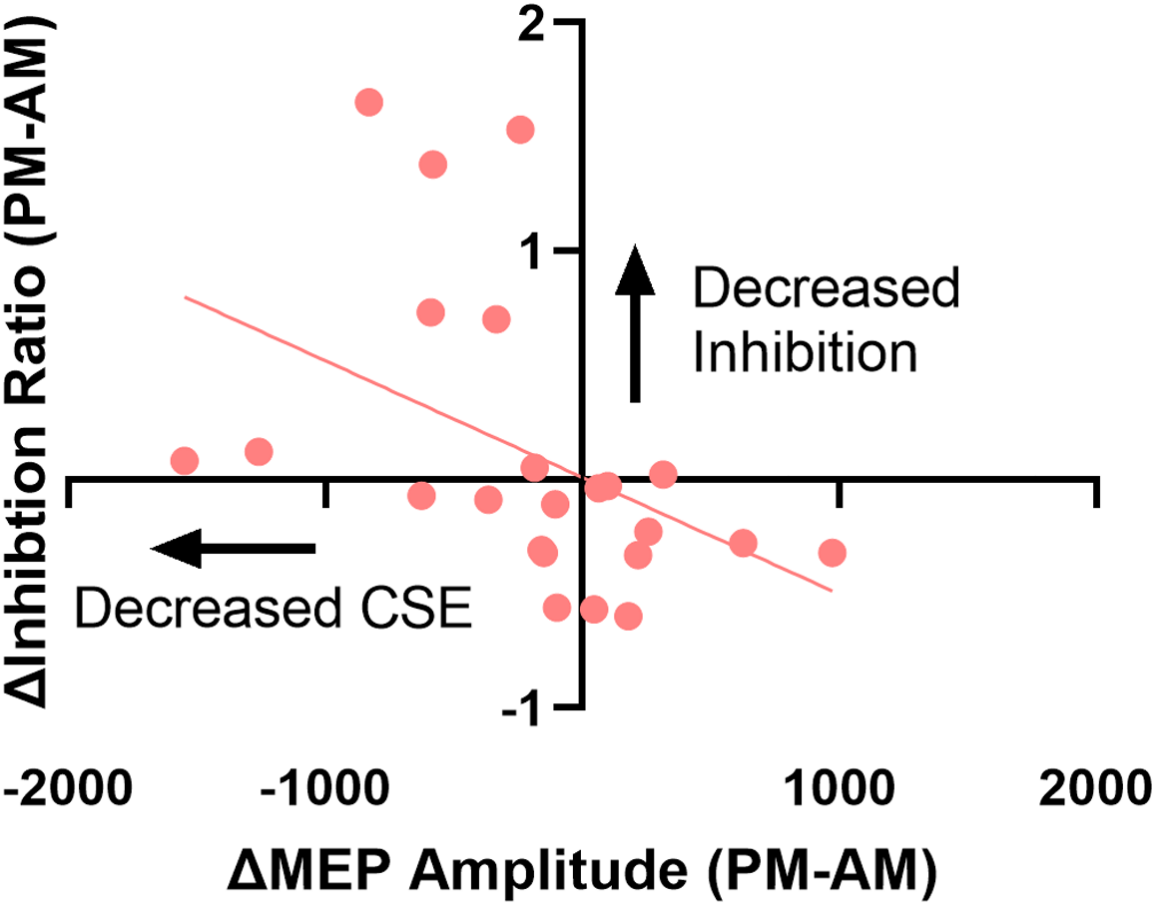
Decreased CSE was significantly associated with decreased SICI in the target hemisphere of participants in the immobilization group (*r* = −0.44, *p* = 0.035). Change in CSE did not correlate with changes in other measures of intracortical or interhemispheric inhibition in either group.

Our results demonstrated that change in SICI was negatively correlated with change in CSE (Pearson's *r* = −0.44, *p* = 0.035) (Figure 5). This correlation indicates that decreased CSE was associated with an index of decreased GABA_A_‐ergic inhibition in the target hemisphere in the immobilization group. There were no significant correlations between change in CSE versus change in LICI (*r* = 0.10, *p* = 0.65), IHI10 (*r* = −0.06, *p* = 0.83), or IHI50 (*r* = −0.04, *p* = 0.86) in the target hemisphere in immobilized individuals. In the control group, change in CSE was not significantly correlated with change in any measure of inhibition (all *p* > 0.05).

## 4 DISCUSSION

Our results demonstrated that 6 h of immobilization decreased CSE in the target hemisphere and increased SIHI from the non‐target to the target hemisphere, in line with previous studies. We also observed that LIHI was increased onto the target hemisphere following immobilization, suggesting that immobilization modulates interhemispheric circuits that involve both GABA_A_ and GABA_B_‐ergic inhibition. However, changes in IHI and CSE were not associated, implying that increased IHI is unlikely the primary driver behind reduced CSE following immobilization. Additionally, our results indicate that immobilization did not significantly modulate intracortical inhibitory circuit activity in the target M1 at the group level. However, reductions in CSE were uniquely associated with reduced intracortical inhibition following immobilization. Taken together, our results demonstrate that increased IHI following short‐term immobilization is likely due to an increase in activity of the excitatory transcallosal projections from the non‐target onto the target hemisphere and may suggest that modulation of intracortical inhibition is related to homeostatic plasticity effects associated with immobilization.

We observed a reduction in CSE due to immobilization of the arm, consistent with other studies (Clark et al., 2010; Crews & Kamen, 2006; Facchini et al., 2002; Huber et al., 2006; Karita et al., 2017; Opie et al., 2016). In the current study, immobilization‐induced decrease in CSE was observed in the largest cohort of participants studied to date and with a duration of immobilization (6 h) shorter than most previous studies, supporting a robust effect of immobilization on reducing CSE. While the assessment of a single TMS intensity (120% RMT) in the current study is a limitation, our results of decreased CSE replicate the results of studies that used more comprehensive input–output curves to characterize CSE (Opie et al., 2016; Rosenkranz et al., 2014). Decreased CSE after immobilization is thought to be due to LTD‐like processes (Huber et al., 2006) and is associated with an enhanced capacity for LTP‐like plasticity after immobilization (Rosenkranz et al., 2014), consistent with the model of homeostatic metaplasticity. TMS measures of CSE are influenced by inhibitory and excitatory circuits in M1 (Ziemann et al., 2015); therefore, characterization of CSE in isolation provides limited insight into circuit‐specific changes in cortical neurophysiology after immobilization.

Characterizing immobilization effects on IHI circuits revealed increased inhibition from the non‐target onto the target hemisphere. Previous studies have found increased SIHI, mediated by GABA_A_, from the non‐target to the target hemisphere after a 10‐h period of immobilization (Avanzino et al., 2011, 2014). Here, were observed a similar effect after a 6‐h period of immobilization. Additionally, we extended previous findings to show that LIHI, primarily thought to be mediated by GABA_B_ receptor activity, also increases onto the target hemisphere. While there was an effect of immobilization on IHI at the group level, the change in IHI after immobilization was not correlated with the change in CSE in the target hemisphere of immobilized participants. This suggests that immobilization influences interhemispheric and corticospinal circuits independently, and decreased CSE after immobilization is not primarily the result of increased IHI from the contralateral hemisphere. The ability of short‐term immobilization to modulate IHI could be leveraged in individuals where a shift in IHI balance would be beneficial. For example, one model of stroke recovery posits that an imbalance in IHI may contribute to persistent motor dysfunction (Di Pino et al., 2014; Di Pino & Di Lazzaro, 2020; Murase et al., 2004). Thus, immobilization may hold promise as a noninvasive neuromodulation approach to rebalance activity between hemisphere to facilitate post‐stroke recovery in some patients. However, it is worth considering that recent evidence suggests that an imbalance in IHI after stroke may be a consequence of changes in behavior rather than a cause (Xu et al., 2019).

Group‐level analyses showed no effect of immobilization on indices of GABA_A_‐ or GABA_B_‐ergic intracortical inhibition. However, in the target hemisphere in immobilized participants, reduced CSE was associated with reduced SICI, an index of intracortical GABA_A_‐ergic inhibition (Ziemann et al., 2015). Importantly, an association between the modulation of CSE and SICI was not present in the target hemisphere of the non‐immobilized control participants, suggesting that the association between reduced CSE and reduced intracortical inhibition was specific to immobilization. The observed relationship with reduced CSE may implicate decreased intracortical inhibition as a potential mechanism for the increase in the capacity for LTP‐like plasticity following immobilization. Previous research has shown that reductions in intracortical inhibition in M1 can facilitate LTP‐like plasticity (Ziemann et al., 2001), while increases in indices of GABA_A_‐ergic inhibition block LTP‐like plasticity in human M1 (Butefisch et al., 2000; Ziemann et al., 2001). In rodent M1, reductions in GABA_A_‐ergic inhibition are required to induce LTP (Hess et al., 1996). Additionally, Rosenkranz et al. (2014) demonstrated that the degree of reduction in SICI was correlated with the amount of LTP‐like plasticity that was able to be induced after 8 h of immobilization, supporting the notion that changes in intracortical inhibition may contribute to homeostatic plasticity mechanisms after immobilization. Of note, there are multiple sets of parameters that can be used to index GABA_A_‐ergic activity, including the parameters used in the current study (Ziemann et al., 2015). It is therefore, possible that the specific parameters chosen for the current study may have influenced our results, and future studies should assess immobilization‐induced changes in multiple indices of GABA_A_‐ergic inhibition.

Taken together, the significant relationship between reduced CSE and reduced SICI may indicate a local reduction in the excitability of intracortical circuits within M1. Our results support the possibility of global downscaling of activity with immobilization, but it should be noted that intracortical inhibition was not significantly modulated by immobilization at the group level. Due to the large inter‐individual variability in neuromodulatory response to short‐term immobilization, further investigation is required to determine the cause of this variability. The high degree of inter‐individual variability could be due, in part, to the use of 120% RMT as the stimulation intensity to measure CSE. To potentially reduce this variability, instead of utilizing 120% RMT, future studies could adjust stimulation intensity to produce MEPs at 1 mV. Similarly, it is possible that collecting recruitment curve data for each of the excitatory and inhibitory measures, rather than stimulation at single intensities relative to motor threshold, would provide a more comprehensive understanding of the effect of immobilization on neural activity. Additionally, future studies should also include evaluation of intracortical facilitatory pathways (e.g., TMS measures of intracortical facilitation [ICF]) to assess whether the downscaling of excitability within M1 is specific to inhibitory circuits or if it is indeed a more global phenomenon.

Changes in the activity of other regions that are interconnected with M1 may also contribute to the decrease in CSE following short‐term immobilization. Given the observation of profoundly reduced movement in the immobilized arm, which necessarily reduces both motor output and sensory input, one potential cortical region of interest is the primary somatosensory cortex (S1). Previous research has demonstrated that M1 and S1 are highly interconnected, and these connections strongly influence motor behavior (Edwards et al., 2019). Only a few studies have examined the effect of immobilization on S1 activity, measuring somatosensory‐evoked potentials (SEPs) in response to peripheral nerve stimulation. Huber et al. (2006) found that after 12 h of arm immobilization, the P45 component of the SEP, thought to reflect activation of S1 (Allison et al., 1992; Sasaki et al., 2018), was reduced in the S1 corresponding to the immobilized arm compared to the non‐immobilized S1. Another study reported an increase in the N9 component, which corresponds to the excitability of the peripheral nerve, after 10 h of arm immobilization (Ikeda et al., 2019). This finding potentially reflects a sensitization of the ascending sensory pathway after short‐term immobilization. However, additional research is necessary to assess if and to what extent these results would be observed following only 6 h of immobilization. The effects of immobilization on other brain regions in the sensorimotor network that have been shown to influence plasticity in human M1, including premotor cortex (Huang et al., 2018), supplementary motor area (Hamada et al., 2009), and cerebellum (Opie & Semmler, 2020), have been less well‐studied but could also influence the targeted neurophysiologic effects observed with TMS‐based measures of M1 excitability.

Measures of spinal excitability were not collected in the current study which may also have influenced TMS‐based assessments of CSE. It has been demonstrated that longer durations of immobilization (e.g., 1 week) led to an increase in spinal excitability (Lundbye‐Jensen & Nielsen, 2008). In contrast, it has been suggested that shorter periods of immobilization, such as hours to days, do not modulate spinal excitability (Bassolino et al., 2012; Facchini et al., 2002). Although participants were provided standardized instructions for the immobilization period and wrist actigraphy demonstrated that the amount of limb activity was substantially reduced during immobilization, individuals were not directly monitored during this period. Given this point, it is possible that the specific activities they engaged in, and the level of engagement, contributed to inter‐individual differences in the neurophysiologic effects of immobilization. It has previously been demonstrated that the amount of use of the non‐immobilized arm can influence changes in intracortical and interhemispheric excitability (Avanzino et al., 2011). Several intraindividual traits, such as attention and genetics, have been shown to influence the capacity for neuroplasticity in human M1 but were not assessed (for a review, see Ridding and Ziemann [2010]). We did not use biological sex as a randomization factor and observed unequal ratios of males to females between groups. Post‐hoc analyses suggest there was a nonsignificant difference between groups in number of males and females (Fisher‐exact *p* = 0.09) and independent samples *t*‐tests demonstrated that there was not a significant difference between males and females in the outcome measures at baseline (all *p* > 0.05). Although, it remains possible that these or other factors may have contributed to inter‐individual variability of the neuromodulatory effects of immobilization.

## 5 CONCLUSIONS

Overall, our results demonstrate that immobilization reduces CSE and increases IHI, consistent with our hypotheses and previous findings. Interestingly, decreased CSE was significantly associated with a decrease in an index of GABA_A_‐ergic intracortical inhibition, suggesting that short‐term arm immobilization may lead to a global downscaling of synaptic strength in M1. Reduced intracortical inhibition may provide a novel mechanism by which short‐term immobilization could enhance the capacity for LTP‐like plasticity within M1 and may be a potential target for strategies to augment plasticity capacity to enhance motor learning in health and disease.

## CONFLICT OF INTERESTS

The authors have no conflicts of interest to disclose.
